# Plant Adaptation to Salinity: Physiological Pathways and Prospects for Crop Improvement—A Review

**DOI:** 10.3390/genes17030253

**Published:** 2026-02-24

**Authors:** Himabindhu Badavath, Christopher A. Saski

**Affiliations:** Department of Plant and Environmental Sciences, Clemson University, Clemson, SC 29634, USA; hbadava@clemson.edu

**Keywords:** salt tolerance, saline soil, genetic mechanisms, CRISPR

## Abstract

Soil salinity is a major abiotic constraint on crop growth, yield, and quality. Advancing salt-tolerant agriculture requires an integrated understanding of salinity-induced osmotic stress, ionic toxicity, and oxidative damage, and how physiological and molecular networks regulate these processes. This review synthesizes key responses to salt stress, including osmotic adjustment, ion transport and compartmentalization, photosynthetic acclimation, reactive oxygen species detoxification, and phytohormone-mediated regulation by integrating mechanistic trial-level and deployment-focused insights while providing a fundamental translational insight into durable crop salt tolerance. We further summarize transcriptional and post-transcriptional control mechanisms involving stress-responsive genes, transcription factor families, and microRNA/non-coding RNA regulation. Finally, we compare progress and constraints in conventional breeding, transgenic approaches, genome editing, and epigenetic strategies for improving salinity tolerance and highlight priorities for translating these mechanistic insights into durable field performance.

## 1. Introduction

Soil salinity reflects the accumulation of soluble salts in the soil solution and is commonly quantified using electrical conductivity. At the plant level, salinity reduces water uptake (osmotic stress) and disrupts nutrient and ion balance (ionic stress), which together limit growth and productivity. With sustained exposure, Na^+^ and Cl^−^ can accumulate in tissues, triggering oxidative stress and downstream metabolic dysfunction [1,2,3].

Soils are commonly classified as saline when the electrical conductivity of the saturated paste extract (ECe) exceeds approximately 4 dS m^−1^ at 25 °C (often reported as a threshold for crop sensitivity), whereas sodic soils are characterized by high exchangeable sodium (e.g., ESP > 15 and/or SAR > 13) and associated physical constraints that may occur with or without high salinity [4]. Because salinity and sodicity can co-occur but act through partially distinct mechanisms, precise terminology is essential for interpreting plant responses and management options. ECe-based values represent soil classification criteria rather than universal crop tolerance thresholds, as salinity sensitivity varies widely among crops, cultivars, developmental stages, and growing environments. Moreover, threshold definitions differ between soil salinity (ECe) and irrigation water salinity (ECw), resulting in distinct exposure dynamics at the root zone that must be considered when interpreting plant responses. At the plant level, salt stress is described as a two-phase process. The initial phase is rapidly driven by osmotic stress, occurring within minutes to hours following exposure and leading to reduced water uptake and transient growth inhibition. A second, slower phase develops over days to weeks as Na^+^ and Cl^−^ progressively accumulate in aerial tissues, where they disrupt cellular metabolism, promote oxidative stress, and impair photosynthetic capacity. The relative contribution of these phases varies depending on the species, genotype, developmental stage, and the intensity and duration of salt exposure.

Salinity impacts multiple developmental stages, including germination, vegetative growth, and reproduction [5]. Photosynthesis is often reduced due to stomatal limitation (restricted CO_2_ diffusion) and direct impairment of chloroplast function, leading to lower biomass accumulation and yield [6,7]. Beyond yield effects, salinity responses in several crops are non-linear, with mild salinity enhancing selected quality traits within a limited range, while higher salinity imposes yield penalties, highlighting threshold-dependent trade-offs.

Global assessments indicate that salt-affected soils are widespread and expanding, and FAO estimates indicate that approximately 1.38 billion hectares (~10.7% of global land area) are affected by salinity in many agricultural regions. The FAO Global Status of Salt-Affected Soils report summarizes large areas of salt-affected land in arid and semi-arid zones [8].

Salinity drivers differ between open-field systems (evaporative demand, drainage constraints, saline irrigation, coastal intrusion) and greenhouse or substrate-based systems, where fertigation and confined root volumes dominate salt dynamics. In irrigated systems, salinity risk is amplified by high evaporative demand, inadequate drainage, and irrigation practices that promote salt accumulation. Coastal agriculture is additionally exposed to saltwater intrusion associated with sea-level rise and storm surges [5,9]. This review synthesizes recent advances in plant salinity tolerance, spanning physiological mechanisms (osmotic adjustment, ion homeostasis, and reactive oxygen species (ROS) detoxification) and their genetic regulation. We also compare the outcomes and limitations of conventional breeding, transgenic approaches, genome editing, and emerging epigenetic perspectives. Finally, we highlight bottlenecks that constrain translation to consistent field performance and outline priorities for integrating mechanistic biology with breeding and deployment. Evidence is organized from genes and pathways to component traits and is then evaluated in the context of deployment across contrasting production environments, with particular emphasis on translational constraints.

## 2. Plant Types and Salt Tolerance Variation

Plants differ widely in salinity tolerance and are often described along a continuum from halophytes (salt-tolerant) to glycophytes (salt-sensitive) [10,11]. Halophytes possess specialized traits that enable growth in saline habitats, whereas most agronomically important crops are glycophytes whose yields decline substantially under elevated salinity [10,11] (Table 1). Tolerance strategies are frequently framed as a combination of (i) Na^+^/Cl^−^ exclusion (restricting entry and/or xylem loading) and (ii) tissue tolerance (vacuolar sequestration, osmotic adjustment, and detoxification of ROS). These strategies are not mutually exclusive and can vary by tissue, developmental stage, and genotype [12,13] (Table 1).

From a crop improvement perspective, the halophyte–glycophyte contrast is most informative when tolerance mechanisms are assessed for genetic feasibility and developmental tractability. Many halophytes depend on anatomically complex traits such as salt glands, very high vacuolar sequestration capacity, or succulence, which are challenging to transfer into crops, whereas mechanisms including Na^+^ exclusion, regulated ion transport, osmotic adjustment, and antioxidant capacity have demonstrated relevance and genetically tractable bases in glycophytes. These mechanisms are also strongly stage- and tissue-dependent: seedling tolerance, vegetative ion homeostasis, and reproductive-stage yield stability are often governed by different processes. This reinforces the need for stage-specific phenotyping and careful target prioritization in breeding programs. Among cereals, rice is frequently regarded as relatively salt-sensitive, whereas barley is comparatively salt-tolerant, making these crops valuable systems for dissecting physiological and molecular mechanisms [14]. In cotton, both interspecific and intraspecific variation influence ion homeostasis, osmotic adjustment, and antioxidant responses, providing additional genetic resources for improving tolerance [15]. During the ionic phase of salt stress, altered accumulation of essential ions (e.g., K^+^, Zn^2+^, Mn^2+^, Mo^2+^) and increased Na^+^/Cl^−^ pressures can disrupt membrane and organelle integrity, reduce nutrient acquisition, accelerate senescence, and impair photosynthesis via chlorophyll degradation and chlorosis in salt-sensitive genotypes [12,16] (Table 1).

## 3. Salt Transport in Plants

Plants absorb water when the root water potential is more negative than that of the surrounding soil. Accumulation of soluble salts, such as Na^+^ lowers soil water potential and creates an osmotic constraint that reduces plant water uptake [13]. The resulting decline in turgor pressure disrupts plant water homeostasis and can reduce water-use efficiency [17,18]. Movement of water and solutes from soil to the root vasculature is constrained by anatomical barriers such as the Casparian strip and associated suberin deposits in the endodermis, which restrict apoplastic transport [13,19]. Consequently, salt entry into roots depends on the pathway program and on transporters and channels in the plasma membranes [20,21].

Salt transport in plants operates across multiple, hierarchically linked spatial scales, encompassing root entry and endodermal restriction, long-distance transport via xylem loading and retrieval that governs shoot ion delivery, and tissue- and cell-level compartmentation that ultimately determines ionic toxicity or tolerance. Variation in salinity tolerance among species primarily reflects differences in the capacity, coordination, and spatial deployment of these processes, rather than the mere presence or absence of individual transporters.

**Figure 1:**
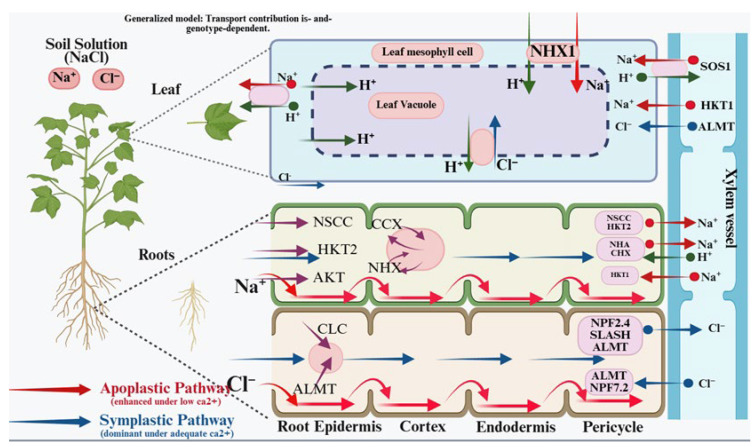
Schematic representation of Na^+^ and Cl^−^ transport pathways across root and leaf tissues. **Abbreviations: NSCC**, non-selective cation channel; **HKT**, high-affinity K^+^ transporter; **AKT**, inward-rectifying K^+^ channel; **NHX/NHA/SOS1**, Na^+^/H^+^ antiport systems; **ALMT**, aluminum-activated malate transporter; **CLC**, chloride channel.

Na^+^ influx can occur through non-selective cation channels (NSCCs) and members of the high-affinity K^+^ transporter family (HKT), among other routes [22,23]. NSCC activity can involve Ca^2+^-regulated channel families such as glutamate receptor-like channels (GLRs) and cyclic nucleotide-gated channels (CNGCs) (Figure 1). Additional contributions to Na^+^ influx and translocation are due to low-affinity cation transporters (e.g., LCT1) and K^+^ channels such as AKT1 [24]. In rice, OsHKT2;1 can facilitate Na^+^ uptake and contribute to seedling sensitivity under salinity [25]. Long-distance Na^+^ transport through xylem and phloem involves transporter families, including HKTs, cation/H^+^/H exchangers (CHXs), and Na^+^/H^+^ antiporters such as SOS1/NHA that participate in Na^+^ extrusion and redistribution [24,26]. Cl^−^ uptake and distribution can involve H^+^/Cl^−^ symporters, Cl^−^/H^+^ co-transporters, nitrate transporters (NRTs), and cation-chloride cotransporters (CCCs). Within cells, Cl^−^ transport and compartmentation are mediated by channels/transporters such as ALMTs and CLCs, including tonoplast-localized components that support vacuolar sequestration (Figure 1).

Transport components vary substantially in the strength of genetic and physiological validation. HKT-mediated xylem Na^+^ retrieval and SOS1-mediated Na^+^ extrusion are supported by strong causal evidence and reproducible phenotypic effects. In contrast, proposed Na^+^ influx pathways associated with non-selective cation channel activity often show substantial redundancy and context dependence, which limits definitive causal attribution in crop systems. In halophytes, salinity tolerance is typically achieved through coordinated regulation of root-level Na^+^ exclusion, controlled long-distance transport, and efficient sequestration of Na^+^ and Cl^−^ within vacuoles or specialized tissues (e.g., epidermal or bladder cells). Together, these mechanisms maintain cytosolic ion homeostasis even when total tissue ion loads are high. In contrast, many glycophytic crops have a more limited capacity for cell-type–specific ion partitioning, leading to greater accumulation of toxic ions in photosynthetically active tissues and heightened sensitivity of growth and reproductive processes under comparable salinity.

A key translational limitation is that transporter-level effects do not scale linearly to whole-plant performance because salinity tolerance depends not only on total ion accumulation, but also on tissue-specific and subcellular localization. Commonly used measurement proxies, such as bulk leaf ion concentrations, leaf sap or xylem sap analyses, and ionomic profiling, integrate signals across compartments and time scales and can obscure cellular partitioning patterns that ultimately govern photosynthetic stability and yield. This mismatch contributes to the frequent divergence between transporter-level phenotypes and agronomic performance under field salinity.

## 4. Salt Stress Sensing and Signaling

Salt stress is perceived at or near the plasma membrane and initiates a network of signal transduction pathways. Early events commonly include changes in ion fluxes (mainly Na^+^ influx), transient cytosolic Ca^2+^ increases, lipid-derived signaling, and the accumulation of ROS [13,27,28]. Cytosolic Ca^2+^ acts as a secondary messenger that is read by Ca^2+^ sensors and kinase modules, including CBL-CIPK complexes, calcium-dependent protein kinases (CDPKs), and MAPK cascades, which together regulate ion homeostasis, osmotic adjustment, and stress-responsive gene expression [29,30]. Kinase-mediated Ca^2+^ and ROS signaling activates key transcription factors, including DREB, NAC, and AREB/ABF, coordinating downstream gene networks that regulate ion balance, support osmotic adjustment, and enhance antioxidant capacity under salt stress. These early signaling events function as integrative nodes that link ionic and osmotic perturbations to downstream processes such as ion transport regulation, hydraulic and stomatal control, ROS homeostasis, and growth modulation. A major outcome of this signaling integration is the activation of regulatory reprogramming factors to stress-responsive gene expression. Framing Ca^2+^ signaling in terms of these physiological outputs provides a clear mechanistic continuity for transcriptional reprogramming, sensing, and subsequent physiological adaptations. A central salt-specific module is the Salt Overly Sensitive (SOS) pathway, in which Ca^2+^ signaling activates SOS2 kinase and the plasma membrane Na^+^/H^+^ antiporter SOS1 to promote Na^+^ extrusion and control long-distance Na^+^ transport [28,31]. In parallel, HKT-mediated Na^+^ retrieval from the xylem can reduce shoot Na^+^ loading and support maintenance of favorable K^+^/Na^+^ ratios [32,33]. The SOS pathway constitutes one of the most causally validated salt-response modules, supported by consistent loss- and gain-of-function genetic evidence with reproducible phenotypic effects. In comparison, other Ca^2+^-dependent kinase networks and MAPK cascades generally display partial redundancy and context dependence, with contributions that vary across tissues, developmental stages, and stress regimes.

Salt stress signaling is connected to abscisic acid (ABA) and other phytohormones. ABA-dependent and ABA-independent pathways jointly shape transcriptional programs that regulate osmolyte accumulation, antioxidant defenses, growth modulation, and ion transport [28,34,35]. Together, these signaling pathways coordinate with stress-responsive gene expression, converging on transcriptional and post-transcriptional regulatory networks.

## 5. Transcriptional and Post-Transcriptional Regulatory Mechanisms Under Salt Stress

In response to salt stress, plants reprogram gene expression through transcription factors (TFs) and post-transcriptional regulators. Major TF families implicated in salt responses include DREB/CBF, NAC, MYB, bZIP, and WRKY, which bind cis-elements in promoters of stress-responsive genes and coordinate downstream pathways such as osmolyte biosynthesis, ROS detoxification, and ion transport [36,37].

Several of these regulatory modules represent conserved nodes validated across crops, including the DREB/CBF regulon and the SOS signaling pathway governing Na^+^ homeostasis. Such conserved regulators are increasingly leveraged in breeding pipelines through marker development, genomic selection frameworks, and targeted genome editing when favorable alleles or limited pleiotropic effects are identified.

Small RNAs, particularly microRNAs, fine-tune mRNA stability and translation, adding an additional layer of regulatory control [38]. However, transcript abundance does not always correlate with protein levels or activity under salt stress due to translational regulation, protein turnover, and post-translational modifications, highlighting a limitation of transcriptome-based analyses. Signal-driven transcriptional changes under salinity contribute to structural remodeling and growth adjustment (e.g., cell wall modification, cytoskeletal reorganization, and hormone-mediated control), and to broad metabolic shifts that support energy balance and stress resilience, including reconfiguration of primary metabolism and enhanced antioxidant capacity [39]. Although these metabolic adjustments improve survival under stress, they can carry substantial carbon and energy costs, as sustained ROS detoxification and osmolyte synthesis may reduce photosynthetic efficiency and, under prolonged salinity, lead to growth or yield penalties. Transcriptional and post-transcriptional regulation mediates the translation of early signaling cues into adaptive physiological traits, linking molecular responses to whole-plant performance.

## 6. Plant Responses and Adaptive Mechanisms Under Salt Stress

The molecular and regulatory adjustments are ultimately reflected in distinct physiological and developmental responses at the whole-plant level under saline conditions. Salinity commonly reduces growth and induces morphological changes, including stunting, delayed organ development, shortened vegetative growth, altered flowering phenology, and accelerated senescence [4,40]. In many crops, shoot growth is more sensitive than root growth, but responses are strongly species- and genotype-dependent [41]. Root system architecture is frequently altered under salinity. In soybean (*Glycine max*), elevated salinity reduces root number and length, constraining water and nutrient uptake [42,43] (Table 2). In wheat (*Triticum aestivum*), saline conditions can reduce total root length, surface area, volume, and lateral root development, with root plasticity contributing to tolerance differences among genotypes [44] (Table 2). This rapid inhibition of root and shoot growth is driven primarily by osmotic stress, making it particularly relevant in short-term assays and early-stage screening. In Arabidopsis, salinity can promote flower bud abscission and impair the development of reproductive organs, including effects on pollen and ovule development [45] (Table 2). In sunflower (*Helianthus annus*), salinity can reduce seed yield and influence oil quality [46]. In tomato (*Solanum lycopersicum*), salinity often reduces fruit weight and yield and can alter quality traits [47] (Table 2). Significantly, mild salinity within a crop-specific threshold can sometimes improve quality traits (e.g., soluble solids or flavor) before yield penalties become apparent. This response is often non-linear, with an “optimal salinity window” in which modest stress enhances specific quality attributes. Beyond this window, yield typically declines rapidly, underscoring the need to distinguish beneficial from detrimental stress intensities and to set cultivar- and environment-specific electrical conductivity (EC) targets for applied systems [48].

At the cellular level, salinity can disrupt chloroplast ultrastructure and thylakoid organization, increasing the risk of photoinhibition and oxidative damage [49]. Plants mitigate these effects through coordinated strategies, including osmoregulation, ROS scavenging, photosynthetic acclimation, phytohormone signaling, and controlled uptake, exclusion, and compartmentalization of ions [12,50]. While osmotic tolerance is critical during early exposure, ionic toxicity driven by Na^+^ accumulation becomes increasingly essential during prolonged or field-level salinity, defining distinct targets for long-term tolerance breeding. Maintenance of a high cytosolic K^+^/Na^+^ ratio is a recurring determinant of tolerance. Na^+^ can displace K^+^ at binding sites and impair enzyme activity; thus, efficient K^+^ uptake and Na^+^ exclusion/sequestration mechanisms support metabolic function under salinity [4,32]. In breeding and phenotyping pipelines, K^+^/Na^+^ ratios are typically measured in specific tissues (e.g., young leaves or shoots), at defined developmental stages, and after sustained exposure. Given that thresholds differ among species and environment-specific variability, standardized sampling is essential for converting molecular and transcriptional responses into actionable phenotypes.

**Table 2:** Representative morphological and yield-related phenotypes reported under salt stress.

Crop/Species	Species Category/Use	Developmental Stage	Trait/Organ	Salinity Level	Experimental System & Stress Type	Representative Phenotype(s) Under Salinity	Mechanism
*Glycine max* (soybean)	Agronomic crop (annual legume)	Seedling stage	Seedling growth and vigor (whole plant)	NaCl treatments with separated Na^+^ and Cl^−^ effects (≈50–150 mM NaCl equivalents)	Controlled nutrient solution; short-term stress	Coordinated inhibition of root elongation, reduced shoot growth and biomass accumulation, leaf chlorosis, and premature senescence, collectively leading to poor seedling establishment.	Cl^−^ toxicity caused greater disruption of ionic homeostasis and osmotic balance than Na^+^; excessive Cl^−^ accumulation impaired water uptake and metabolic activity more severely than Na^+^ at comparable salinity levels [42,43].
*Glycine max*, *Glycine soja*, and interspecific hybrid	Crop, wild relative, and hybrid (annual legumes)	Seedling stage	Root and shoot tissue ion fluxes	50–150 mM NaCl	Controlled hydroponic system; short-term stress	*G. soja* exhibited higher salt and Cl^−^ tolerance than *G. max*; hybrids showed intermediate tolerance with reduced growth inhibition	Salt tolerance is associated with lower Na^+^ and Cl^−^ accumulation in sensitive tissues and more stable cellular ion fluxes, notably improved Cl^−^ regulation [43].
*Triticum aestivum* (wheat)	Agronomic crop (annual cereal)	Seedling stage	Root system architecture, shoot thermal profile, and ion homeostasis	Incremental short-term NaCl stress (≈50–150 mM NaCl)	Controlled conditions; incremental short-term stress	Reduced total root length, surface area, and volume, and altered lateral root development; genotypic differences in root plasticity were observed.	Tolerance linked to improved Na^+^ exclusion, higher K^+^ retention, stable Na^+^/K^+^ ratios, and adaptive root architectural responses supporting water uptake [44].
*Arabidopsis thaliana*	Model organism (annual dicot)	Vegetative to reproductive transition	Reproductive development	Moderate–high NaCl stress (≈100–200 mM NaCl)	Controlled growth conditions; short-term to moderate stress	Flower bud abscission and impaired reproductive organ development under salinity.	Osmoprotection via glycine betaine accumulation stabilized cellular membranes and proteins under salt stress (example: transgenic enhancement of glycine betaine biosynthesis) [45].
*Brassica napus* (canola)	Agronomic crop (annual oilseed)	Seedling and vegetative stages	Growth, chlorophyll content, oxidative stress markers	Soil salinity (≈6 dS m^−1^ NaCl equivalent)	Soil-based pot experiment; chronic salinity	Reduced shoot and root growth, decline in chlorophyll content, and increased lipid peroxidation; antioxidant application mitigated growth inhibition and oxidative damage.	Salt stress induced excess ROS production leading to membrane lipid peroxidation; antioxidant application improved ROS scavenging and maintained photosynthetic pigments [47].
*Helianthus annuus* (sunflower)	Agronomic crop (annual oilseed)	Reproductive/seed-filling stage	Seed yield and oil quality	≈4–12 dS m^−1^ (field-relevant salinity levels)	Field or soil-based conditions; chronic salinity	Decreased oleic acid (C18:1) and increased linoleic acid (C18:2) during seed filling; altered fatty acid composition reflected salinity-induced metabolic shifts.	Yield reduction linked to impaired assimilate partitioning and reproductive development; salinity altered lipid biosynthesis via modulation of desaturase activity [46].
*Punica granatum* (pomegranate)	Perennial fruit crop	Vegetative growth	Whole-plant growth; tissue ion distribution	60 mM and 120 mM NaCl	Nutrient solution culture; short-term to moderate stress	Reduced biomass without severe leaf necrosis; increased Na^+^ and Cl^−^ accumulation in roots and stems; reduced K^+^ and Ca^2+^ in leaves and stems; altered micronutrient levels.	Ionic imbalance disrupted K^+^/Na^+^ and Ca^2+^/Na^+^ ratios; Ca^2+^ supplementation partially mitigated stress via membrane stabilization and reduced Na^+^ translocation [51].

Footnote: Salinity levels are reported as mM NaCl in solution culture or as electrical conductivity (dS m^−1^) under soil conditions; direct quantitative comparisons across systems should be interpreted cautiously.

## 7. Conventional Selection and Breeding Strategies for Salinity Tolerance

Although conventional breeding has delivered significant yield gains in the twentieth century, improving abiotic-stress tolerance has often progressed more slowly due to polygenic inheritance, strong genotype-by-environment interactions, and trade-offs between stress tolerance and yield potential [52]. Breeding programs have exploited genetic variation at intra-specific and inter-specific levels to develop salt-tolerant lines and cultivars (Table 3**)**. In alfalfa (*Medicago sativa* L.), germplasm lines released through conventional selection showed improved germination under high-salt conditions and modest forage yield gains in early field trials [53]. In cotton, early physiological and field-based screening studies revealed substantial variation in salinity responses among *Gossypium* accessions, which formed the basis for selection-based improvement [54]. Similarly, in tomato, wild relatives such as *Solanum pimpinellifolium* were identified early as salt-tolerant germplasm and used for introgression into cultivated backgrounds [55] (Table 3).

In conventional breeding, salinity tolerance is typically polygenic, involving many small-effect loci that collectively regulate ion exclusion (e.g., HKT-type transporters), tissue tolerance, osmotic adjustment, and yield stability, rather than single significant genes. Best-practice breeding, therefore, combines multi-environment trials with selection in relevant stress environments to capture genotype-by-environment interactions and to prioritize traits relevant to specific production systems and growth stages. However, donor-based introgression is often constrained by linkage drag, genetic background dependence, and long breeding cycles, and even when physiological improvements are achieved, potential yield or quality penalties and the cost of field validation remain significant barriers to practical adoption.

**Table 3:** Conventional selection and breeding strategies for improving salinity tolerance in major crop species.

Crop (Species)	Salt-Tolerant Donor	Target Trait(s) (Mechanistic)	Breeding Approach/Notes	Salinity Level & Experimental System	Outcome	Limitations/Critical Assessment
Wheat (*Triticum aestivum*)	Kharchia-65 (derived from Kharchia landrace)	Whole-plant salinity tolerance under sodic–saline soils; yield stability; ion exclusion–linked adaptation	Conventional selection and crossing/backcrossing into adapted backgrounds; backbone donor for Indian salt-tolerant wheat (e.g., KRL1-4 = Kharchia-65 × WL711)	Farmer fields with sodic–saline soils in Rajasthan; subsequent testing across saline soils of North India (field-chronic stress)	Kharchia-65 became the primary donor for Indian salt-tolerant wheat; KRL1-4 performed well in North India	Strong background dependence and G × E effects; poor performance in Pakistan highlights linkage drag and environment-specific expression; limited mechanistic resolution delayed gene-level deployment [56]
Wheat (*T. aestivum*)	Kharchia-65 × WH1105 (recurrent elite parent)	Na^+^ exclusion–associated tolerance; germination and multi-trait performance under salinity	SSR-based marker-assisted backcross breeding targeting *Nax1*/*Nax2* with background recovery	Controlled chloride-dominated salinity (ECe ≈ 8 dS m^−1^); seedling screening in trays followed by pot growth to maturity	Demonstrated faster recovery of elite background while retaining tolerance loci	Screening is confined mainly to controlled conditions; yield stability under heterogeneous saline–sodic and waterlogged field environments remains insufficiently validated [56]
Rice (*Oryza sativa*)	Pokkali; Nona Bokra	Seedling-stage salinity tolerance; Na^+^ exclusion; low shoot Na^+^/K^+^ ratio; survival (SES scoring)	Conventional donor-parent breeding using standardized IRRI screening protocols	Controlled hydroponic/nutrient-solution screening; EC ≈ 12 dS m^−1^ (seedling stage)	Pokkali and Nona Bokra became global donor standards	Narrow donor base; significant loci (Saltol/*OsHKT1;5*) explain key components but not complete tolerance; seedling tolerance often fails to predict reproductive-stage yield [57,58,59].
Rice (*O. sativa*)	Pokkali somaclonal variants (e.g., CSR10)	Salinity tolerance with improved plant type	Somaclonal variation via tissue culture regeneration followed by physiological screening	Hydroponic screening and field evaluation under saline conditions	CSR10 is reported as an improved salt-tolerant cultivar	Genetic basis difficult to track and pyramid; somaclonal variation limits predictability and long-term transferability, motivating a shift toward QTL/gene-tagged introgression [60].
Tomato (*Solanum lycopersicum*)	*Solanum pennellii* (wild relative)	Osmotic tolerance; reduced Na^+^ accumulation; partial ion homeostasis	Introgression breeding using defined introgression line populations	Controlled salinity screening (germination and vegetative stages)	Improved individual component traits	Yield gains constrained by trade-offs and G × E interactions; introgressed segments often carry adverse agronomic effects (linkage drag) [61,62].
Cotton (*Gossypium* spp.)	*G. barbadense* accessions; salt-tolerant *G. hirsutum* landraces	Germination, growth, boll retention, and fiber yield under moderate salinity	Hybridization and recurrent phenotypic selection with elite *G. hirsutum* backgrounds	Controlled NaCl-amended soils (EC ≤ ~12 dS m^−1^) and multi-season field trials on naturally saline soils (ECe ≈ 5–8 dS m^−1^)	Development of cotton lines with improved growth and fiber yield under moderate salinity	Quantitative tolerance with slow genetic gain; strong background effects and long breeding cycles limit rapid deployment [63].

## 8. Genetic Engineering and Transgenic Approaches

Transgenic studies have validated roles for ion transporters, transcription factors, and antioxidant pathways in salinity tolerance, although performance in the field can be variable and environment dependent. A prevalent strategy is the overexpression of ion transporters and proton pumps that support Na^+^ efflux and/or vacuolar sequestration. For example, co-expression of wheat TaNHX1 and TVP1 enhanced vacuolar Na^+^ and K^+^ storage in Arabidopsis, improving tolerance to salinity [64]. Heterologous expression of wheat TaNHX2 improved salt tolerance in transgenic tomato by enhancing vacuolar Na^+^ sequestration [65]. At the plasma membrane, SOS1-mediated Na^+^/H^+^ exchange, coordinated with H^+^-ATPase activity, supports Na^+^ extrusion and reduces shoot Na^+^ accumulation [66]. Maintaining elevated K^+^/Na^+^ ratios by combining K^+^ channel regulation with HKT-mediated Na^+^ retrieval from the xylem provides another route to tolerance [32]. Transcription factor engineering has also shown promise. For instance, overexpression of stress-responsive NAC family members has been associated with enhanced salinity tolerance in wheat and related cereals, consistent with a broader role for NAC TFs in stress adaptation [67]. Field performance of transgenic salinity traits is often inconsistent for several well-defined reasons. Constitutive overexpression of ion transporters (e.g., NHX or SOS1) can impose energetic costs, perturb K^+^ homeostasis, and interfere with developmental and reproductive programs. Using stress- or tissue-specific promoters can mitigate these penalties, but such constructs often produce more minor or variable effects. In addition, improved ion homeostasis, such as reduced shoot Na^+^ or higher K^+^/Na^+^ ratios, does not consistently translate into biomass or yield gains, because compensatory trade-offs in growth and fertility frequently emerge. These limitations underscore the need to evaluate candidate traits using whole-plant performance metrics, not ion balance alone.

## 9. CRISPR and Genome Editing Technologies

Genome editing enables targeted modification of endogenous loci to alter salinity-relevant traits and, in some cases, can avoid stable integration of foreign DNA. In soybean, CRISPR/Cas9 knockout of the GmAITR genes produced mutants with elevated leaf K^+^/Na^+^ ratios and improved salt tolerance, validating transcriptional regulators as actionable targets [68] (Table 4). In rice, CRISPR/Cas9 knockout of OsCIPK9 enhanced salt tolerance, supporting signaling components that control ion homeostasis as candidate loci for improvement [69] (Table 4). Beyond gene knockouts, promoter editing and precision nucleotide modification (base and prime editing) can generate favorable alleles while preserving native expression patterns. Multiplex editing offers opportunities to stack complementary component traits (e.g., root architecture, osmotic adjustment, and ROS detoxification) to improve whole-plant performance [70,71] (Table 4).

Genome-editing targets for salinity tolerance can be classified into functionally distinct classes, providing a structured framework for systematic evaluation beyond single-gene examples. These classes encompass (i) ion transporters and channels mediating Na^+^ exclusion and compartmentation (e.g., HKT and SOS components), (ii) signaling regulators, including kinases and phosphatases, that control ion homeostasis, (iii) transcription factors and repressors coordinating stress-responsive gene networks, and (iv) hormone biosynthesis and signaling genes, particularly those involved in ABA-mediated osmotic adjustment. Additional target categories include genes involved in osmolyte biosynthesis, antioxidant-mediated ROS detoxification, water transport regulation (aquaporins), cell wall modification, and negative regulators of stress tolerance that are well-suited to loss-of-function editing. Organizing genome-editing targets into functional categories clarifies how specific edits contribute to distinct tolerance components, including Na^+^ exclusion, tissue tolerance, osmotic adjustment, and sustained growth under salinity.

Notably, most reported transgenic and CRISPR-based salinity tolerance gains have been validated under controlled conditions, commonly using acute NaCl treatments at seedling or early vegetative stages. Field validation conditions with chronic or spatially heterogeneous salinity remain scarce, and improvements in ion balance or stress traits often fail to confer stable reproductive-stage yield. Multiplex genome editing enables integration of complementary tolerance components, yet effective stacking depends on explicit prioritization and validation. Rational design strategies typically emphasize (i) Na^+^ exclusion to minimize shoot ion toxicity, (ii) tissue tolerance and osmotic adjustment, and (iii) root architectural or water transport traits supporting sustained uptake under salinity. Adequate validation of multiplex editing staged, from individual edits to multi-gene combinations across stages and environments.

**Table 4:** CRISPR-validated and candidate genome-editing targets for improving salinity tolerance in plants.

Crop (Species)	Objective	Gene/Pathway	Editing Strategy	Validation Level	Salinity Level & Conditions	Impact and Significance
Rice (*Oryza sativa*)	Shoot Na+ exclusion/reduced shoot Na+ loading	OsHKT1;5 (Saltol locus; xylem Na+ transporter)	Proposed allele-specific or promoter/cis-regulatory editing	Field-validated locus (breeding-relevant); CRISPR editing proposed	Moderate–high salinity widely used across studies (often ~80–120 mM NaCl in hydroponic system; soil-based validation also reported in genetic/physiological studies; chronic vs. short-term varies by study)	Natural allelic variation enhances xylem Na+ retrieval, limiting shoot Na+ loading; high-confidence locus for precise editing deployment [58].
Wheat (*Triticum aestivum*)	Shoot Na+ exclusion + yield stability under chronic salinity	TaHKT1;5-D (Kna1 locus; Na+ transporter)	Proposed allele editing/elite allele deployment	Field-validated locus (chronic salinity); CRISPR editing proposed	Saline soil + controlled salt treatments reported; chronic field-relevant exposure emphasized in breeding/physiology	Limits shoot Na+ accumulation; supports improved ionic balance and grain yield under salinity; cornerstone locus for translational improvement [72].
Rice (*Oryza sativa*)	Ion-homeostasis signaling modulation	OsCIPK9 (CBL-interacting protein kinase; interacts with OsSOS3)	CRISPR/Cas9 knockout	Greenhouse-validated (controlled conditions); CRISPR knockout demonstrated	Salt treatments in controlled assays (paper reports salt-responsive expression and KO improving tolerance; conditions vary by study)	KO improves salt tolerance; supports CIPK nodes as actionable signaling targets [69].
Rice (*Oryza sativa*)	Seedling tolerance via regulator removal (negative regulator KO)	OsRR22 (regulator of salt response)	CRISPR/Cas9 knockout (frameshift)	Greenhouse-validated (seedling stage); CRISPR knockout demonstrated	~100–150 mM NaCl; hydroponic/controlled; typically, acute-to-short term at seedling stage	Knockout mutants show enhanced seedling tolerance; transgene-free segregants reported; agronomic penalties not emphasized in early generations [73].
Arabidopsis (*A. thaliana*, model)	Relief of ABA-mediated repression (negative regulators)	AITR family (ABA-induced transcription repressors)	CRISPR/Cas9 multiplex family knockout	Model species; greenhouse-validated; CRISPR multiplex knockout demonstrated	Controlled salinity assays: stress regimes reported in the primary CRISPR study	Cas9-free quintuple/sextuple mutants show enhanced salt (and drought) tolerance and reduced ABA sensitivity; supports knockout of negative regulators [74].
Rice (*Oryza sativa*)	ABA pathway tuning for osmotic adjustment	OsPYL9 (ABA receptor; PYR/PYL family)	Proposed CRISPR fine-tuning (expression/allele modulation)	Lab-level functional evidence; salinity-specific CRISPR validation proposed	Controlled stress assays: NaCl levels not standardized across reports	OsPYLs characterized; OsPYL9 functional work supports ABA-receptor tuning as a plausible route; needs salt-specific genome-editing validation [75].
Rice (*Oryza sativa*)	Cis-regulatory editing (method exemplar; not salinity target)	Xa13 promoter (SWEET-related susceptibility context)	CRISPR/Cas9 promoter editing	Greenhouse-validated (methodological exemplar; non-salinity context)	Controlled conditions; disease-resistance context (not a salinity phenotype)	Demonstrates promoter editing as a precise, transgene-free strategy applicable to stress genes with expression trade-offs [76].
Tomato (*Solanum lycopersicum*)	SOS signaling to maintain Na+/K+ homeostasis	SlSOS2 (SlCIPK24)	Transgenic overexpression (editing proposed)	Greenhouse-validated (transgenic overexpression); genome editing proposed	~100–150 mM NaCl; controlled; often chronic vegetative assays	Overexpression improves Na+ exclusion/ionic balance and salt tolerance; supports SOS nodes as conserved improvement targets; genome editing proposed [77].

Footnote: Reported impacts are based on direct genome-editing validation were indicated or on established genetic and physiological evidence supporting these loci as candidates for editing; validation categories in the table reflect the highest level of experimental or field evidence currently available and do not imply equivalent translational readiness.

## 10. Epigenetic Regulation and Stress Memory

Epigenetic mechanisms influence chromatin accessibility and transcriptional capability, enabling dynamic regulation of stress-responsive genes without changing DNA sequence. Key mechanisms include DNA methylation, histone modifications/variants, and non-coding RNAs (miRNAs, siRNAs, and lncRNAs). Studies in rice and other cereals indicate that salinity can be associated with altered DNA methylation and histone modification patterns that correlate with differential gene expression and cultivar-specific tolerance, and in some cases contribute to stress ‘memory’ that primes responses to recurring stress [78,79,80,81]. Non-coding RNAs provide additional regulatory layers. For example, osa-MIR393 is responsive to salinity and targets stress-associated genes, and its overexpression can increase stress sensitivity [38]. In cotton, the salt-induced lncRNA lncRNA973 is upregulated under salinity and has been reported to act as a positive regulator of tolerance [82]. However, epigenetic and transcriptomic analyses provide valuable insights into stress-responsive genes and may not reliably predict protein-level function or physiological responses due to incomplete correlation between mRNA and protein abundance, and post-transcriptional and post-translational regulation can further modulate functional outcomes under salinity, highlighting the incomplete correlation between transcript abundance and actual protein activity. More broadly, evidence for salinity-induced stress memory is primarily limited to short-term within-generation priming or, at most, limited mitotic inheritance. Stable meiotic transmission across generations appears rare and is often inconsistent across studies. The reproducibility and persistence of epigenetic states depend strongly on genetic background, developmental stage, and environmental context, and many stress-induced marks are at least partially reset during reproduction, restricting their reproducibility and direct deployment in breeding programs.

## 11. Conclusions and Future Perspectives

Salt stress typically occurs during an early osmotic phase, followed by a progressive ionic phase, together driving coordinated transcriptional, metabolic, and physiological changes that determine growth and yield outcomes [4,12]. Tolerance depends on integrating water status control, ion homeostasis, and ROS management across tissues and development. Conventional breeding remains foundational but is constrained by quantitative inheritance and genotype-by-environment effects [83]. Transgenic studies and genome editing have validated key pathways (ion transport, signaling, and regulatory networks) and offer opportunities to engineer favorable alleles directly in elite backgrounds [50,68].

Looking ahead, the next generation of salt-tolerant crops will require (i) robust, scalable phenotyping that captures whole-plant performance under realistic field salinity; (ii) deeper mechanistic resolution of tissue- and stage-specific tolerance components; (iii) strategic stacking of complementary traits (e.g., ion exclusion combined with tissue tolerance and osmotic adjustment); and (iv) integrated use of genomics and epigenomics to accelerate selection and deployment. Progress will depend on tightly coupling mechanistic discovery to breeding pipelines and validating outcomes in target environments. Crucially, “agronomically relevant salinity” differs across production systems. In open-field and irrigated agriculture, salinity is typically chronic and spatially heterogeneous, shaped by irrigation water quality, evaporative demand, soil properties, and drainage constraints rather than short-term, acute salt exposure. In greenhouse and substrate-based systems, salinity is driven primarily by fertigation practices, recirculating nutrient solutions, and limited leaching capacity, which can constrain the feasibility of implementing genotype-specific electrical conductivity targets at a commercial scale. Accordingly, genetic improvement will be insufficient; effective deployment will depend on scalable phenotyping and compatibility with existing irrigation and drainage infrastructure, water quality limitations, and the practical realities of integrating tolerant cultivars into current production systems. Thus, effective crop salinity tolerance improvement will require systematic, stage-specific phenotyping and environment-specific selection and deployment strategies; mechanistic advances translate into reliable performance under real-world agricultural conditions.

## Figures and Tables

**Table 1:** Key physiological and molecular differences between halophytes and glycophyte crops relevant to salinity tolerance and breeding.

Feature	Halophytes	Glycophyte Crops	Breeding Relevance
Na^+^ handling	Salt glands, extreme sequestration	HKT/SOS-mediated exclusion	High (HKT, SOS loci)
Tissue tolerance	Succulence, salt dilution	Limited vacuolar capacity	Moderate
Developmental control	Often constitutive	Strong stage dependence	High (phenotyping critical)
Genetic tractability	Often complex/polygenic	Several causal loci are known	High
Yield under salinity	Maintained	Often stage-limited	Primary breeding bottleneck

## Data Availability

No new data were created or analyzed in this study. Data sharing is not applicable to this article.
